# Aminopeptidase N-null neonatal piglets are protected from transmissible gastroenteritis virus but not porcine epidemic diarrhea virus

**DOI:** 10.1038/s41598-019-49838-y

**Published:** 2019-09-12

**Authors:** Lei Luo, Shaohua Wang, Lin Zhu, Baochao Fan, Tong Liu, Lefeng Wang, Panpan Zhao, Yanna Dang, Pei Sun, Jianwen Chen, Yunhai Zhang, Xinjian Chang, Zhengyu Yu, Huanan Wang, Rongli Guo, Bin Li, Kun Zhang

**Affiliations:** 1 Laboratory of Mammalian Molecular Embryology, College of Animal Sciences, Zhejiang University, Hangzhou, Zhejiang 310058 China; 2 Institute of Veterinary Medicine, Jiangsu Academy of Agricultural Sciences, Key Laboratory of Veterinary Biological Engineering and Technology, Ministry of Agriculture, Nanjing, Jiangsu 210014 China; 3 Jiangsu Co-innovation Center for Prevention and Control of Important Animal Infectious Diseases and Zoonoses, Yangzhou University, Yangzhou, Jiangsu 225000 China; 4 College of Animal Science and Technology, Anhui Agricultural University, Hefei, Anhui 230036 China; 5 School of Food and Biological Engineering, Jiangsu University, Zhenjiang, 212013 China

**Keywords:** Biological techniques, Cloning

## Abstract

Swine enteric diseases have caused significant economic loss and have been considered as the major threat to the global swine industry. Several coronaviruses, including transmissible gastroenteritis virus (TGEV) and porcine epidemic diarrhea virus (PEDV), have been identified as the causative agents of these diseases. Effective measures to control these diseases are lacking. The major host cells of transmissible gastroenteritis virus and porcine epidemic diarrhea virus have thought to be epithelial cells on small intestine villi. Aminopeptidase-N (APN) has been described as the putative receptor for entry of transmissible gastroenteritis virus and porcine epidemic diarrhea virus into cells *in vitro*. Recently, Whitworth *et al*. have reported that APN knockout pigs are resistant to TGEV but not PEDV after weaning. However, it remains unclear if APN-null neonatal pigs are protected from TGEV. Here we report the generation of APN-null pigs by using CRISPR/Cas9 technology followed by somatic cell nuclear transfer. APN-null pigs are produced with normal pregnancy rate and viability, indicating lack of APN is not embryonic lethal. After viral challenge, APN-null neonatal piglets are resistant to highly virulent transmissible gastroenteritis virus. Histopathological analyses indicate APN-null pigs exhibit normal small intestine villi, while wildtype pigs show typical lesions in small intestines. Immunochemistry analyses confirm that no transmissible gastroenteritis virus antigen is detected in target tissues in APN-null piglets. However, upon porcine epidemic diarrhea virus challenge, APN-null pigs are still susceptible with 100% mortality. Collectively, this report provides a viable tool for producing animals with enhanced resistance to TGEV and clarifies that APN is dispensable for the PEDV infection in pigs.

## Introduction

Swine enteric coronavirus disease is a devastating disease that has caused significant economic losses to the global swine industry^1^. Transmissible gastroenteritis virus (TGEV) and porcine epidemic diarrhea virus (PEDV) have traditionally been the major etiological agents of swine enteric diseases, in particular, in Asia. Since 2010, greater economic losses have been made due to the outbreak of emerging and reemerging CoVs, including variant strains of PEDV^2^, porcine deltacoronavirus (PDCoV) and swine acute diarrhea syndrome coronavirus (SADS-CoV)^3–5^. Only in year 2013, PEDV outbreak has wiped out more than 10% of America’s pig population^5^. These CoVs primarily affect suckling piglets and result in high morbidity and up to 100% mortality. They infects the small intestine and can induce watery diarrhea and vomiting, eventually leading to dehydration, body weight loss, and other devastating outcomes. To date, no effective means are available to control these diseases.

Receptor binding and cellular entry are crucial for understanding of the infection processes, host range and tropism of a virus as well as identifying targets for antiviral interventions^6^. For example, CD163 has been identified as an entry receptor for porcine reproductive and respiratory syndrome (PRRS) virus and subsequent studies demonstrated that CD163-null pigs are immune to PRRS, which presents a valuable resource for breeding PRRS-resistant pigs^7^. Aminopeptidase-N (APN) is widely present in the membrane of the epithelial cells of the small intestine in a variety of species, including pigs. In addition, it has been shown to be involved in multiple functions, including peptide metabolism, cell adhesion and cholesterol uptake^8^. Curiously, APN has also been suggested as a receptor for multiple alphacoronaviruses, including TGEV^9^, PDCoV^10,11^, and human coronavirus 229E (HCoV-229E)^12,13^. However, these reports relied on the use of cell line or other *in vitro* models, raising the concern regarding the biological significance of APN’s role *in vivo*. Meanwhile, whether APN is also required for PEDV infection is unresolved. Previous studies regarding of the role of APN in PEDV susceptibility have yielded conflicting results^14–19^. To resolve unambiguously the functional requirement for porcine APN in TGEV and PEDV infection *in vivo*, APN-null pigs have to be produced and viral challenge assay performed. Recently, Whitworth *et al*. have reported APN-null post-weaning pigs are resistant to TGEV but not PEDV^20^. However, TGEV and PEDV primarily affect suckling piglets with up to 100% mortality and post-weaning pigs have been shown to be less susceptible to these viruses^21^. We believe it is more biological relevant to determine if APN-null neonatal piglets (<10 days) are resistant to TGEV and PEDV. Meanwhile, only 1 APN-null piglet was used for PEDV challenge experiment in Whitworth *et al*. study, further consolidating the necessity to clarify the role of APN in PEDV infection again.

Here, we generated APN-null pigs using CRISPR/Cas9 and somatic cell nuclear transfer (SCNT) technology. After viral challenge, the neonatal pigs exhibited significant resistance to TGEV infection. However, APN-null neonatal pigs are still susceptible to PEDV infection. Together with Whitworth *et al*.’s work, the present study highlights the potential for APN editing in swine breeding to improve TGEV resistance. The report also clarify that APN is dispensable for PEDV infection in pigs.

## Results

### Bi-allelic modification of APN in porcine fetal fibroblast cells by using CRISPR/Cas9 double nicking nuclease

**Figure 1:**
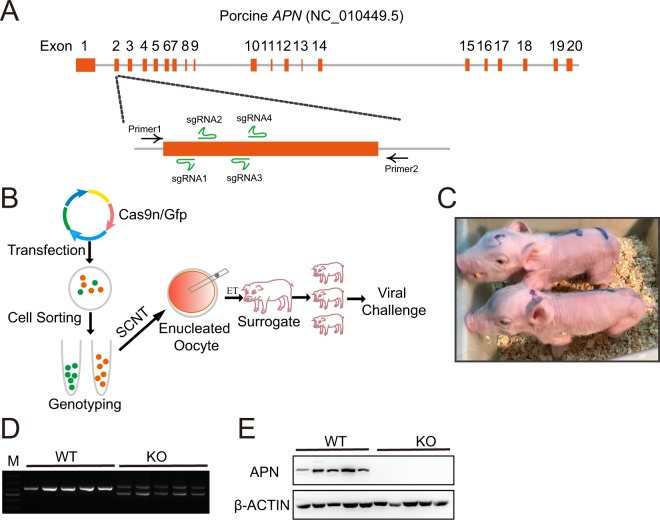
Efficient generation of APN-null pigs using a combination of CRISPR/Cas9 and somatic cell nuclear transfer technology. (**A**) Schematic description of APN genome structure and the targeting strategy. Exons of APN are shown as orange, and exon 2 represents the sgRNA targeting site, which is enlarged to a rectangle box. Primer 1/Primer 2 are designed for genotyping in (**D**). (**B**) Schematic overview of the production of APN-null pigs. (**C**) Representative APN-null pigs at day 2. (**D**) PCR genotyping-confirmed site-specific modification at APN in the cloned pigs. Sequencing results were shown as Table S3. Lanes 1–5 represent 5 wildtype individuals and lanes 6–10 represent 5 APN-null pigs (KO1-5), full-length gels are presented in Supplementary Fig. 3A. (**E**) Western blot analysis confirmed absence of APN in the APN-null pigs (*n* = 5). Full-length blots are presented in Supplementary Fig. 3B,C.

A CRISPR/Cas9 double nicking nuclease (Cas9n) editing system was used to disrupt porcine APN in the present study given the presumable potential of Cas9n in reducing off-target activity^22^. To target the endogenous APN for inactivation, a combination of two pairs of CRISPR small guide RNA (sgRNA) specific to the second exon was designed and predicted to introduce early indel mutations to the APN genomic sequence (Fig. 1A). To maximize the cutting efficiency at the target site, a single plasmid based on PX461 was constructed and encoded a pair of sgRNAs driven by U6 promoter, and Cas9-2A-GFP driven by CAG promoter.

The schematic for the generation of APN-null pigs is presented in Fig. 1B. The plasmid containing Cas9n and sgRNA was transfected into porcine fetal fibroblast cells. Two days later, fluorescence activated cell sorting (FACS) was performed to isolate transfected cells, which were cultured into a 100 mm plate with 50–100 cells per dish. To improve the growth performance of the transfected single cells, basic Fibroblast Growth Factor (FGF2) was supplemented into the culture medium. Transfected cells were harvested and genotyped by PCR and subsequent sequencing. It was found that 16 out of 19 cell colonies (84.2%) were identified as bi-allelic modification at *APN*, suggesting a robust editing efficiency. Transfected cells were used as donor cells for somatic cell nuclear transfer (SCNT).

### Generation of APN knockout pigs via somatic cell nuclear transfer

Initial attempts to produce APN-null pigs have been inefficient with only 2 viable piglets out of 6 embryo transfers (150 embryos/transfer), which may due to the inherent limitation of the particular donor cell line used. To overcome the limitation, a pool of transfected cells or fibroblast cell lines established from the APN-null pig were used as donors for subsequent SCNT experiments. Results showed a normal early embryogenesis with above 40% of constructed APN-null embryos developing into blastocyst stage after being cultured for 6 days *in vitro* (Table S1). A total of 1,866 SCNT embryos were transferred into the oviducts of 10 surrogate sows in two days with one week apart. We detected a pregnancy rate of 70% (d50) and all these pregnancies went to term (Table S2). Twenty-one female piglets were born by natural delivery from seven litters with normal SCNT efficiency (1.13%) and the representative piglets are shown in Fig. 1C. Litter size ranged from two to four. Eight piglets failed to thrive within 2 days after birth because of low birth weight, which was associated with the SCNT. All the remaining piglets are healthy prior to viral challenge. Genotyping of small intestines revealed that all the SCNT piglets exhibited bi-allelic modifications at the genomic DNA level (Fig. 1D represents K1-K5 and Table S3 are sequencing results of all cloned piglets used in the present study) and predicted to be translational knockout (KO) (*APN*^−/−^). Furthermore, APN KO was confirmed by quantitating protein expression (Fig. 1E) in small intestine in the APN-null piglets. Taken together, these data suggest APN is dispensable for embryogenesis and fetal development in pigs. This conclusion maybe not surprising given that APN-null mice also develop normally without physiological changes^23^.

### Gene-edited pigs are protected from transmissible gastroenteritis virus

APN has been demonstrated as the entry receptor for TGEV infection *in vitro*^9,10^. Thus, we hypothesized that APN-null pigs are resistant to TGEV infection. To test the hypothesis, in Experiment 1, colostrum-deprived APN-null pigs (n = 2) together with wild-type (WT) piglets (n = 5) at 2 days of age matched by breed (Large White by Landrace cross) and body weight were orally inoculated with a highly virulent TGEV JS2012 strain. All WT pigs developed typical clinical signs of TGEV infection, including severe watery diarrhea, transient vomiting and lethargy by 36 h post-inoculation (hpi) and 4 of them died by 72 hpi. The onset of these clinical signs is consistent with the detection of stool virus shedding in WT piglets on 24–48 hpi as determined by qPCR (Fig. S1B) and ELISA assay (Table S4). In contrast, both APN-null piglets (n = 2) showed no evidence of diarrhea by 72 hpi. One of the APN-null piglets was euthanized at 96 hpi due to body weakness that may result from insufficient food intake and colostrum deprivation. The remaining pigs were euthanized on 144 hpi.

In order to monitor further stages of infection in detail if APN-null pigs really infected with TGEV during viral challenge and if TGEV resistance is APN indel pattern-dependent, in Experiment 2, APN-null piglets (n = 4) with three distinct APN modifications (Table S3) and WT piglets (n = 5) were challenged with TGEV and sacrificed for pathological examination at 2 stages of infection: acute and mid stages (<48 h, and >48 h, respectively). All piglets of Experiment 1 and 2 were necropsied (K16 and K17 (APN-null pigs ID): 24 hpi; K18 and K20: 48 hpi; K5: 96 hpi; K4: 144 hpi; W8 and W20 (wildtype pigs ID): 24 hpi; W7, W10, W17, W18 and W19: 48 hpi; W9 and W16: 72 hpi; W6: 144 hpi).

Gross pathological analysis showed that all WT pigs exhibited intestinal swelling, yellow stench fluid-filled small intestines with transparent walls, and the distended and bleeding stomach filled with curdled and undigested milk (Fig. S1A). However, APN-null pigs show normal morphology regardless of the indel patterns at *APN* (Fig. S1A).

**Figure 2:**
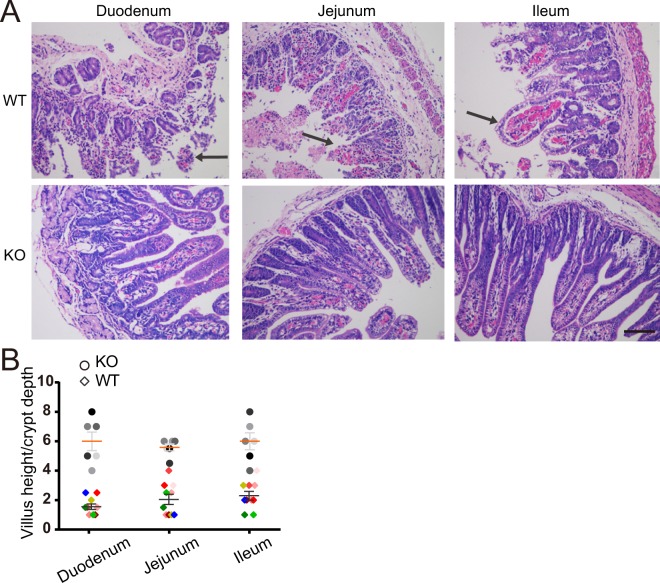
APN-null pigs maintain normal small intestinal architecture upon TGEV challenge. (**A**) Representative pictures of hematoxylin and eosin-stained tissue from wild-type (WT) and APN-null piglets upon necropsy. Histological lesions, including villus atrophy and vacuolation (arrows), were obvious in WT pigs, but not in APN-null pigs. Scale bar = 50 μm. (**B**) The ratio of villus height to crypt depth was strikingly higher in APN-null pigs (*n* = 6) than those of WT pigs (*n* = 10). Each dot represent one pig. Sampling time were: K16 and K17 (APN-null pigs ID): 24 hpi; K18 and K20: 48 hpi; K5: 96 hpi; K4: 144 hpi; W8 and W20 (wildtype pigs ID): 24 hpi; W7, W10, W17, W18 and W19: 48 hpi; W9 and W16: 72 hpi; W6: 144 hpi. Data in (**B**) were shown as mean ± SEM and were analyzed with student’s *t* test. **Denotes significant differences (*P* < 0.05).

The pathological changes seen in WT pigs are accompanied by substantial histological changes in the small intestine. Histopathological analysis confirmed that WT pigs displayed severe necrosis and villous atrophy of the duodenum, jejunum, and ileum, and vacuolation of small intestinal epithelial cells (Fig. 2A). Contrastingly, no obvious intestinal lesions were found in APN-null piglets (Fig. 2A). The mean villous height/crypt depth (VH/CD) ratio of the small intestines of APN-null piglets was significantly greater than that in the WT piglets (Fig. 2B).

**Figure 3:**
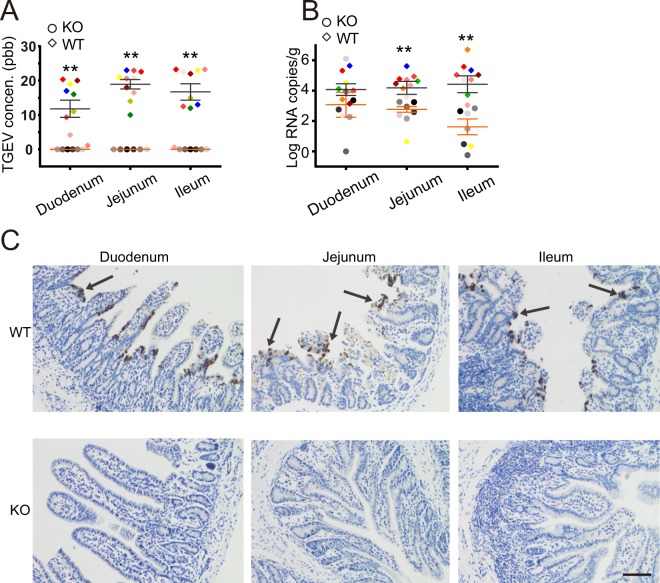
TGEV antigen distribution in small intestines collected from wild-type and APN-null piglets upon TGEV challenge. (**A**) Viral antigen was quantified in pig tissues by ELISA. TGEV antigen concentration is higher in all segments of small intestine from APN-null pigs (*n*  V6) than from wildtype (WT) pigs (*n* (W10). (**B)** TGEV viral RNA genome copies in tissues were quantified by real-time PCR in WT (*n* = 8 for duodenum; *n* = 10 for both jejunum and ileum) and APN-null pigs (*n* = 6 for all sections). For panel A and B, Each dot represent one pig. (**C**) Immunohistochemistry (IHC) analysis of small intestinal tissues (Duodenum, Jejunum, Ileum, *n* = 10 for WT and *n* = 6 for APN-null pigs). Black arrows refer to positive antigens. Sampling time for A-C were: K16 and K17 (APN-null pigs ID): 24 hpi; K18 and K20: 48 hpi; K5: 96 hpi; K4: 144 hpi; W8 and W20 (wildtype pigs ID): 24 hpi; W7, W10, W17, W18 and W19: 48 hpi; W9 and W16: 72 hpi; W6: 144 hpi. Scale bar = 50 μm. Data in (**A,B**) are shown as mean ± SEM. **Indicate significant differences (*P* < 0.05).

To further determine the viral load in gastrointestinal tissues, ELISA assay showed TGEV were readily detected in all sections of small intestine (duodenum, jejunum, and ileum; Fig. 3A). Nonetheless, TGEV was barely seen in tissues of APN-null piglets. In addition, qPCR showed TGEV RNA is relatively lower (in particular in jejunum and ileum) in APN-null than WT piglets (Fig. 3B). Thus, these data clearly suggest that APN is necessary for TGEV infection *in vivo*. Additionally, immunohistochemistry (IHC) analysis confirmed the presence of TGEV antigen in the epithelial cells of atrophied villi in all segments of the small intestines in WT piglets (Fig. 3C). However, APN-null piglets showed no evidence of TGEV-positive cells (Fig. 3C). Thus, we conclude APN is required for TGEV infection *in vivo* in pigs.

### APN-edited pigs are not protected from porcine epidemic diarrhea virus

**Figure 4:**
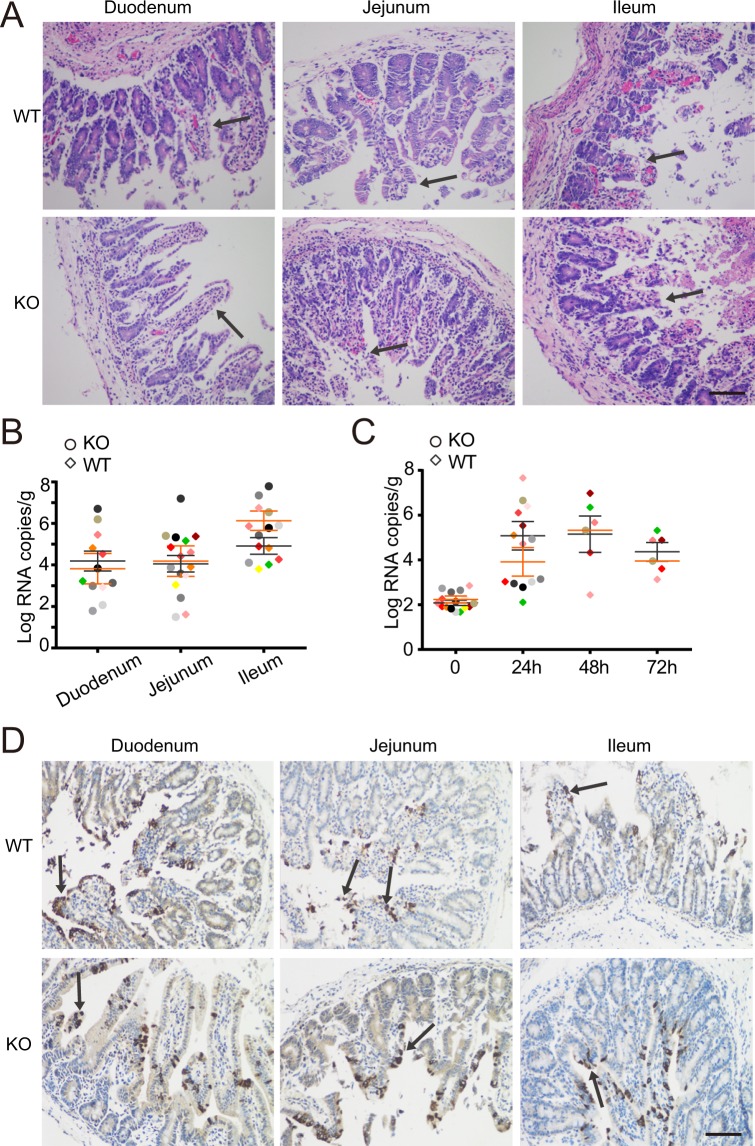
APN is dispensable for PEDV infection in pigs. (**A**) Representative pictures of hematoxylin and eosin-stained tissue from wild-type (WT) and APN-null pigs after PEDV challenge. Histological lesions (arrows) were clearly observed in both WT pigs and APN-null pigs. Scale bar = 50 μm. (**B**) PEDV viral RNA was quantified in pig tissues by real-time PCR. PEDV RNA copies in all segments of small intestine are similar between APN-null pigs (*n* = 7) and WT pigs (at least 5 piglets were analyzed for all sections). Each dot represent one pig. Sampling time for A and B were: K11 and K12: 24 hpi; K1, K2, K3 and K14: 48 hpi; K19: 72 hpi; W13 and W14: 24 hpi; W1 and W4: 48 hpi; W12 and W15: 72 hpi; W2, W3 and W5: 96 hpi. (**C**) PEDV viral RNA genome copies in fecal samples were quantified by real-time PCR. (**D**) Immunohistochemistry (IHC) analysis of small intestinal tissues (Duodenum, Jejunum, Ileum, *n* = 10 for WT and *n* = 7 for APN-null pigs). Arrows indicate positive antigens. Data in (**B**,**C**) are shown as mean ± SEM.

Whether APN is the entry receptor for PEDV remains controversial. To resolve the paradox on the requirement of APN in PEDV infection, APN-null piglets were challenged with PEDV, which caused huge economic loss globally in recent years. All WT (n = 10) and APN-null (n = 7) piglets displayed lethargy and severe watery diarrhea by 24 hpi and died by 84 hpi. Meanwhile, the onset of these clinical signs was accompanied with the detection of PEDV in fecal samples between 24–72 hpi as determined by qPCR (Fig. 4C) and ELISA (Fig. S2D).

All WT and APN-null piglets displayed typical PED clinical lesions, including watery contents-filled and thin-walled small intestines (Fig. S2A). Furthermore, we observed histological changes, including villous atrophy of the enterocytes in all segments of small intestines in both WT and APN-null piglets (Fig. 4A; K11 and K12: 24 hpi; K1, K2, K3 and K14: 48 hpi; K19: 72 hpi; W13 and W14: 24 hpi; W1 and W4: 48 hpi; W12 and W15: 72 hpi; W2, W3 and W5: 96 hpi). The mean ratios of VH/CD for the small intestines were similar between APN-null and WT piglets (Fig. S2B). ELISA and qPCR assays both indicate PEDV was present at a similar level in the small intestines of both WT and APN-null piglets (Figs 4B and S2C). IHC results showed PEDV antigen is detected mainly in the epithelial cells on atrophied villi in all segments of the small intestines of both WT and APN-null piglets (Fig. 4D). Overall, these data indicate APN may be dispensable for PEDV infection *in vivo*.

## Discussion

APN is a multifunctional protein that is involved in the regulation of multiple biological processes, including peptide metabolism, cell adhesion and CoV entry. We have deleted the entire protein in the present study, which may impede physiological functions of APN. Nonetheless, no obvious phenotypic changes have been found with our APN-null pigs with normal birth rate and viability. Moreover, APN knockout in mice results in “no gross or histological abnormalities” while “standard neurological, cardiovascular, metabolic, locomotor, and hematological studies revealed no alterations”^23^. Structural analyses have suggested that the recognition sites for CoV are located on the exposed outer surface of APN and CoV binding does not interfere with other functions of APN^8^. Future work should be directed toward modifying only the critical binding site of APN with CoVs while maintaining other biological functions of APN. One such example is the production of CD163 modified pigs, for which only the exon responsible for PRRSV recognition is disrupted. Such CD163 pigs are resistant to PRRSV and appears normal for other biological performance^24^.

Our studies establish that pig APN is required for TGEV infection. Nonetheless, we could not rule out the possibility of residual TGEV infection in the absence of APN in pigs, suggesting that additional cellular factors contribute to the binding and entry of TGEV. A recent study indeed demonstrated that epidermal growth factor receptor (EGFR) is another receptor critical for TEGV entry^25^.

Results presented herein clarified that APN may be not essential for PEDV infection via genome-modified animal model. Results suggest other cellular receptors should be responsible for PEDV infection. Indeed, PEDV could infect Vero (African monkey kidney) cells, which do not express APN^17^. Additionally, complete absence of human APN in Huh-7 cells or pig APN in ST cells do not affect PEDV infection in these cells^18^. The advent of genome-wide CRISPR-Cas9 library screen in PEDV-susceptible cell lines may provide a viable tool to identify the critical functional receptor for PEDV infection^26^.

The present study evaluates only limited number of APN-modified animals and a single strain for TGEV and PEDV. Additionally, APN has recently been characterized as an entry receptor for PDCoV^10,11,27^, a novel globally distributed CoV identified in 2012^4^. Clearly, it warrants further investigation of other TGEV and PEDV variants as well as PDCoV. For potential agricultural applications, effects of APN-editing have also to be substantially evaluated in large-scale pig populations on phenotypes related to production efficiency, including growth performance and feed conversion efficiency.

Recently, Whitworth *et al*. have also showed APN-null pigs are resistant to TGEV but not PEDV after weaning. However, given we have completed this work independently, there are at least three major differences between our studies. First, only 1 APN knockout pig was used for PEDV challenge experiment in Whitworth’s study compared to 7 in our study. Second, we performed viral challenge experiment in colostrum-deprived neonatal piglets instead of post-weaning piglets used in Whitworth’s study. Since TGEV and PEDV primarily affect suckling piglets with up to 100% mortality and post-weaning pigs have been shown to be less susceptible to these viruses and often survive^21^, we believe the animal model we used is more biological relevant. Third, a CRISPR/Cas9 double nicking nuclease (Cas9n) editing system was used to disrupt porcine APN in the present study given the reduced off-target activity of Cas9n^28^ compared with Cas9 in Whitworth’s study.

In summary, we generate APN-null pigs via genome editing and reproductive cloning. Our results provide a clear role for APN in TGEV infection and suggest that APN may be dispensable for PEDV infection in neonatal pigs. Use of this genetic model is not only valuable in exploring further the role of APN in other biological functions but potentially reduce economic losses related to TGEV in swine industry.

## Methods

### Plasmids

To construct the targeting vector expressing Cas9n and one pair of sgRNA, each oligonucleotide encoding sgRNA was ligated with Bbs-digested PX461 plasmid (Gfp-containing) to construct PX461-sgRNA1 and PX461-sgRNA2, in which sgRNAs are driven by U6 promoters. U6-sgRNA2 was amplified by PCR, digested with Kpn I (New England Biolabs), and fused with Kpn I-treated PX461-sgRNA1 (New England Biolabs). The resultant plasmid was named PX-461-A213 + A258. The sequence of the four oligonucleotides encoding sgRNAs (Shanghai Sangon) were: sgRNA1 (A213-Fwd): caccgCAGGCAACAGCGTTGTGGGT; sgRNA2 (A213-Rev): aaacACCCACAACGCTGTTGCCTGc; sgRNA3 (A258-Fwd): caccgACCCTACCTCACTCCCAACG; sgRNA4 (A258-Rev): aaacCGTTGGGAGTGAGGTAGGGTc.

### Cell Transfection and Culture

Porcine fetal fibroblast cells (Large White by Landrace) were cultured and transfected as previously described with minor modifications. Briefly, PX-461-A213 + A258 was transfected into cells using Lipofectamine 2000 (Invitrogen). Two days after transfection, cells were collected and subjected to FACs sorting to purify GFP-positive cells. Transfected cells were cultured in medium containing 15% fetal bovine serum and 2.5 ng/mL basic fibroblast growth factor (Gibco) in 100 mm culture dishes with 50–100 cells/dish for 9–12 days. The medium was changed every 3 d. Upon confluent, single cell colonies were frozen and genotyped by PCR and sequencing.

### Somatic cell nuclear transfer and embryo transfer

Oocyte maturation, nuclear transfer, and embryo transfer were performed as described previously^29,30^. Briefly, ovaries were obtained from a local abattoir and transported to the laboratory within 2 h in a physiological saline at 37 °C. Cumulus-oocyte complexes (COCs) were aspirated from follicles with 3–6 mm in diameter. Only COCs with 2–3 layers intact cumulus cells were selected for maturation. After 40–42 h, the oocytes with extruded first polar bodies were used for subsequent nuclear transfer. The nuclear transfer, fusion and activation of reconstructed embryos were conducted as published previously. All reconstructed embryos were cultured in the porcine zygote medium-3 (PZM-3) for 20–24 h until embryo transfer at 38.5 °C under 5% CO_2_. As quality controls, approximately 20 reconstructed embryos were cultured *in vitro* for 7 days to monitor their developmental potential to reach blastocyst stage, 40–70 embryos were parthenogenetically activated and cultured to control the oocyte quality. Embryos were transferred into the oviduct of surrogates on the first day of standing estrus. Pregnancies were examined by ultrasound at day 28 after embryo transfer. All SCNT pigs were delivered naturally.

### Genotyping

Genomic DNA was extracted from the ear tissue of cloned piglets using phenol-chloroform approach. Amplification of *APN* was performed using the forward primer 5′-GGGATATAAGCCTGGTCCGAAG-3′ and reverse primer 5′-AAGTTCCCCCTGGAATTCACTC-3′, and preparation of reaction system based on the manual with the AccuPrimeTM Taq DNA Polymerase, Hight Fidelity (Invitrogen). PCR conditions were 94 °C for 15 s, followed by 35 cycles of 94 °C for 15 s, 58 °C for 15 s, 68 °C for 1 min and a final extension of 72 °C for 5 min. The PCR product was separated on 2% agarose gel and the genotype determined by Sanger sequencing.

### Cells and virus isolates

Vero and ST cells were cultured in Dulbecco’s Modified Eagle’s Medium (DMEM; Life Technologies, Carlsbad, CA, USA) supplemented with antibiotics (100 units/mL of penicillin, 100 mg/mL of streptomycin, and 0.25 mg/mL of fungizone) (Life Technologies), and 10% heat-inactivated fetal bovine serum (Life Technologies). The Vero-81 (ATCC No. CCL-81) cell line was used for PEDV propagation and titration. TGEV was propagated in ST cells. PEDV AH2012/12 stain (Gene bank No. KU646831) has been described in previous study (Fan *et al*., 2017). TGEV JS2012 (Genebank No. KT696544) was isolated in Dr. B. Li lab. The isolates used in this study were confirmed negative for PDCoV and porcine rotaviruses.

### Viral challenge and sample collection

Two or three-day-old colostrum-deprived piglets were subject to viral challenge. The piglets were fed with liquid milk replacer every 3 hours. All piglets were randomly divided into TGEV and PEDV challenge experiment and housed in separate rooms. Prior to viral challenge, the piglets were confirmed negative for RNA and antibody of PEDV and TGEV. Piglets were challenged orally with 1 mL PEDV AH2012/12 with a titer of 10^5.0^ TCID_50_, or 1 mL TGEV JS2012 with a titer of 10^6.0^ TCID_50_. After challenge, the piglets were monitored and evaluated daily for clinical signs and rectal swab samples were collected. Rectal swabs were collected for enumerating fecal viral RNA shedding by quantitative real-time PCR. Upon necropsy, intestinal tissues were grossly evaluated. Duodenum, jejunum and ileum tissues were fixed in 10% formalin for histopathology and immunohistochemistry examinations.

### Viral real-time quantitative PCR (RT-qPCR)

The viral RNAs in stomach and small intestinal samples were quantified by RT-qPCR in our laboratory as published previously^31^. Briefly, total RNAs were extracted using Rneasy Mini Kit (Qiagen), and reverse transcription was conducted using the SuperScript III First-Strand Synthesis Kit (Invitrogen). Taqman qPCR was performed against TGEV or PEDV in a 96-well optical plate (Applied Biosystems) at 95 °C for 10 min, followed by 40 cycles of 95 °C for 30 s, 60 °C for 30 s, and 72 °C for 30 s. The sequences of primers and probes for TGEV and PEDV^31^ were listed as following: TGEV: forward: 5′-CCCGTGGTCGGAAGAGTAATAA-3′; reverse: 5′-GGGTACAAAGTCTCTCGGACATAAG-3′; TaqMan probe: 5′-TCTTTCATTCTTCAACCCCATAACCCTCCA-3′; PEDV: forward: 5′-CGCAAAGACTGAACCCACTAACTT-3′, reverse: 5′-TTGCCTCTGTTGTTACTCGGGGAT-3′, probe: 5′-TGTTGCCATTGCCACGACTATAC-3′. The purified PEDV and TGEV genomic RNAs were used to generate a standard curve in RT-qPCR assays.

### ELISA assay

The viral concentrations in fecal, stomach and small intestinal (duodenum, jejunum and ileum) samples were determined by ELISA. The fecal, stomach and small intestinal samples were homogenized in PBS and subject to repeated freezing and thawing in liquid nitrogen. The homogenate was centrifuged at 12,000 g for 10 min at 4 °C. The viral concentrations in the supernatants were quantified by double antibody sandwich ELISA. Briefly, microplates were coated with 100 μl/well of the capture mAb against PEDV or TGEV at a concentration of 2 μg/mL at 4 °C overnight. A blocking buffer (150 μl/well of 1% bovine serum albumin) was added to the plate for 3 h at 37 °C. After three washes with PBST, the supernatants of collected samples and the serially diluted recombinant PEDV-N or TGEV-N protein standard were added into the wells in duplicate, and the plates were incubated at 25 °C for 45 min. After three washes with PBST, HRP-labeled polyclonal antibodies against PEDV or TGEV (100 μl/well) were added at a working concentration with 2 μg/ml, and plates were incubated at 25 °C for 45 min. Then, the wells were washed, followed by the addition of TMB solution (100 μl/well). The reaction was terminated by the addition of sulfuric acid (0.3 N, 100 μl/well) 15 min later. The absorption was measured at 450 nm using a microplate reader, and the virus concentrations were calculated according to the standard curve.

### Western blotting

Ileum samples were lysed on ice in RIPA lysis buffer (Beyotime) supplemented with 1 mM phenylmethylsulfonyl fluoride (Beyotime). The protein concentration was measured using BCA Protine Assay Kit (Beyotime). Protein were separated by 8% SDS-PAGE and transferred to a polyvinylidene fluoride membrane (Millipore). Then, membrane was blocked with 5% non-fat milk and incubated with primary antibodies overnight at 4°C and secondary antibodies for 1.5 h at room temperature. Signals were detected with WESTAR NOVA 2.0 (Cyanagen). The antibodies used were: rabbit anti-APN (1:2000, a generous gift from YW Huang^11^), mouse anti-β-actin (1:2000, Beyotime), HRP-conjugated anti-mouse IgG (1:10000, A0216; Beyotime) or HRP-conjugated anti-rabbit IgG (1:10000, A0208; Beyotime).

### Hematoxylin and eosin staining, and immunohistochemistry

Fresh small intestinal (duodenum, jejunum and ileum) tissues were excised and fixed in 4% PFA and dehydrated overnight in 70% ethanol. The fixed specimens were embedded in paraffin, cut into 5 μm-thick sections and stained with hematoxylin and eosin using a standard protocol. The distribution of TGEV and PEDV antigen was determined by immunohistochemistry with a TGEV and PEDV-specific monoclonal antibody, which was produced in our laboratory using the antigen retrieval method described previously^32^. Samples were observed by conventional light microscopy.

### Statistical analysis

The statistical data were analyzed from at least three biological replicates. Results are stated as the mean ± SEM. Differences between two groups were determined by Student’s *t* tests.

### Ethics approval

All experiments involving animals were performed based on the guidelines for the care and use of lab animals and approved by the Animal Ethics Committee of Jiangsu Academy of Agricultural Sciences (NKYVET 2014-063).

## Supplementary information

Sup infor

## References

[1] Chattha KS, Roth JA, Saif LJ (2015). Strategies for design and application of enteric viral vaccines. Annu Rev Anim Biosci.

[2] Huang, Y. W. *et al*. Origin, Evolution, and Genotyping of Emergent Porcine Epidemic Diarrhea Virus Strains in the United States. *Mbio* **4**, 10.1128/mBio.00737-13 (2013). 10.1128/mBio.00737-13 PMC3812708 24129257

[3] Ma, Y. M. *et al*. Origin, Evolution, and Virulence of Porcine Deltacoronaviruses in the United States. *Mbio* **6**, 10.1128/mBio.00064-15 (2015). 10.1128/mBio.00064-15 PMC4453528 25759498

[4] Woo PC (2012). Discovery of seven novel Mammalian and avian coronaviruses in the genus deltacoronavirus supports bat coronaviruses as the gene source of alphacoronavirus and betacoronavirus and avian coronaviruses as the gene source of gammacoronavirus and deltacoronavirus. Journal of virology.

[5] Mole B (2013). ANIMAL DISEASE Deadly pig virus slips through US borders. Nature.

[6] Li F (2015). Receptor Recognition Mechanisms of Coronaviruses: a Decade of Structural Studies. Journal of virology.

[7] Whitworth KM (2016). Gene-edited pigs are protected from porcine reproductive and respiratory syndrome virus. Nat Biotechnol.

[8] Chen L, Lin YL, Peng G, Li F (2012). Structural basis for multifunctional roles of mammalian aminopeptidase N. Proc Natl Acad Sci USA.

[9] Delmas B (1992). Aminopeptidase-N Is a Major Receptor for the Enteropathogenic Coronavirus Tgev. Nature.

[10] Li WT (2018). Broad receptor engagement of an emerging global coronavirus may potentiate its diverse cross-species transmissibility. Proc Natl Acad Sci USA.

[11] Wang, B. *et al*. Porcine Deltacoronavirus Engages the Transmissible Gastroenteritis Virus Functional Receptor Porcine Aminopeptidase N for Infectious Cellular Entry. *Journal of virology* **92**, 10.1128/JVI.00318-18 (2018). 10.1128/JVI.00318-18 PMC5974500 29618640

[12] Yeager CL (1992). Human Aminopeptidase-N Is a Receptor for Human Coronavirus-229e. Nature.

[13] Lassnig C (2005). Development of a transgenic mouse model susceptible to human coronavirus 229E. Proc Natl Acad Sci USA.

[14] Park JE (2015). Development of transgenic mouse model expressing porcine aminopeptidase N and its susceptibility to porcine epidemic diarrhea virus. Virus Res.

[15] Li BX, Ge JW, Li YJ (2007). Porcine aminopeptidase N is a functional receptor for the PEDV coronavirus. Virology.

[16] Liu C (2015). Receptor Usage and Cell Entry of Porcine Epidemic Diarrhea Coronavirus. J Virol.

[17] Ji CM, Wang B, Zhou JY, Huang YW (2018). Aminopeptidase-N-independent entry of porcine epidemic diarrhea virus into Vero or porcine small intestine epithelial cells. Virology.

[18] Li W (2017). Aminopeptidase N is not required for porcine epidemic diarrhea virus cell entry. Virus Res.

[19] Shirato K (2016). Porcine aminopeptidase N is not a cellular receptor of porcine epidemic diarrhea virus, but promotes its infectivity via aminopeptidase activity. J Gen Virol.

[20] Whitworth KM (2019). Resistance to coronavirus infection in amino peptidase N-deficient pigs. Transgenic Res.

[21] Xia, L., Dai, L., Yu, Q. & Yang, Q. Persistent Transmissible Gastroenteritis Virus Infection Enhances Enterotoxigenic Escherichia coli K88 Adhesion by Promoting Epithelial-Mesenchymal Transition in Intestinal Epithelial Cells. *Journal of virology* **91**, 10.1128/JVI.01256-17 (2017). 10.1128/JVI.01256-17 PMC5640843 28794036

[22] Ran FA (2013). Double nicking by RNA-guided CRISPR Cas9 for enhanced genome editing specificity. Cell.

[23] Rangel R (2007). Impaired angiogenesis in aminopeptidase N-null mice. Proc Natl Acad Sci USA.

[24] Burkard C (2017). Precision engineering for PRRSV resistance in pigs: Macrophages from genome edited pigs lacking CD163 SRCR5 domain are fully resistant to both PRRSV genotypes while maintaining biological function. PLoS pathogens.

[25] Hu WW, Zhang S, Shen YM, Yang Q (2018). Epidermal growth factor receptor is a co-factor for transmissible gastroenteritis virus entry. Virology.

[26] Zhang R (2018). Mxra8 is a receptor for multiple arthritogenic alphaviruses. Nature.

[27] Zhu Xinyu, Liu Shudan, Wang Xunlei, Luo Zhaochen, Shi Yuejun, Wang Dang, Peng Guiqing, Chen Huanchun, Fang Liurong, Xiao Shaobo (2018). Contribution of porcine aminopeptidase N to porcine deltacoronavirus infection. Emerging Microbes & Infections.

[28] Ran FA (2013). Double Nicking by RNA-Guided CRISPR Cas9 for Enhanced Genome Editing Specificity (vol 154, pg 1380, 2013). Cell.

[29] Ding B (2017). WDR5 in porcine preimplantation embryos: expression, regulation of epigenetic modifications and requirement for early developmentdagger. Biology of reproduction.

[30] Tao J (2017). DOT1L inhibitor improves early development of porcine somatic cell nuclear transfer embryos. PloS one.

[31] Fan B (2017). Characterization of a pathogenic full-length cDNA clone of a virulent porcine epidemic diarrhea virus strain AH2012/12 in China. Virology.

[32] Madson DM (2014). Pathogenesis of porcine epidemic diarrhea virus isolate (US/Iowa/18984/2013) in 3-week-old weaned pigs. Veterinary microbiology.

