# Supplementary material for: Aminopeptidase N-null neonatal piglets are protected from transmissible gastroenteritis virus but not porcine epidemic diarrhea virus
Source: Sci Rep. 2019 Sep 12;9:13186. doi: 10.1038/s41598-019-49838-y (PMC6742759; doi:10.1038/s41598-019-49838-y)
Supplement: Supplementary file 1 — Sup infor [file 41598_2019_49838_MOESM1_ESM.pdf]

Aminopeptidase N-null neonatal piglets are protected from transmissible gastroenteritis virus but not porcine epidemic diarrhea virus

Lei Luo[a,d,1], Shaohua Wang[a,1], Lin Zhu[b,1], Baochao Fan[b], Tong Liu[a], Lefeng Wang[a], Panpan Zhao[a], Yanna Dang[a], Pei Sun[d], Jianwen Chen[d], Yunhai Zhang[d], Xinjian Chang[b], Zhengyu Yu[b], Huanan Wang[a], Rongli Guo[b], Bin Li[b,c,2], and Kun Zhang[a,2]

Figure Legends

Fig. S1. APN-null piglets are resistant to TGEV infection. (A) Macroscopic examination of TGEV-challenged wild-type (WT) and APN-null (KO) piglets. (B) TGEV viral RNA genome copies in fecal samples were quantified by real-time PCR.

Fig. S2. APN-null piglets are still susceptible to PEDV infection. (A) Macroscopic examination of PEDV-challenged wild-type (WT) and APN-null (KO) piglets. (B) The ratio of villus height to crypt depth was similar between APN-null pigs (n=7) and WT pigs (n=10). (C and D) PEDV antigen was quantified in pig tissues (C) and fecal samples (D) by ELISA. Data in (B-D) were shown as mean ± SEM.

Table S1.

Summary of somatic cell nuclear transfer results using APN-null porcine fibroblast cells as donors.

Table S2.

Summary of embryo transfer experiments from APN-null cloned embryos.

Table S3.

Genotypes of cloned piglets from APN-null porcine fibroblast cells. K1-K3, K11, K12, K14 and K19: 7 APN-null piglets used for PEDV challenge experiments. K4, K5, K16, K17, K18 and K20: 6 APN-null piglets used for TGEV challenge experiments.

Table S4.

Fecal shedding in TGEV-challenged wildtype (WT) and APN-null (KO) piglets.

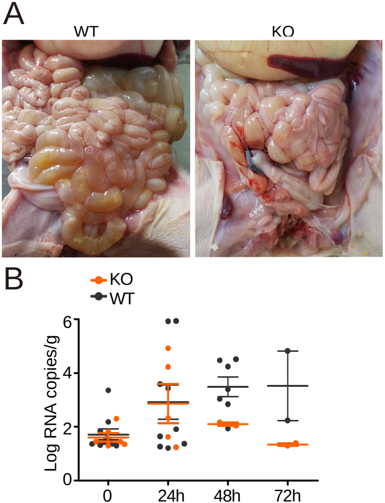
A
WT
KO
B
KO
WT
Log RNA copies/g
0
2
4
6
0
24h
48h
72h

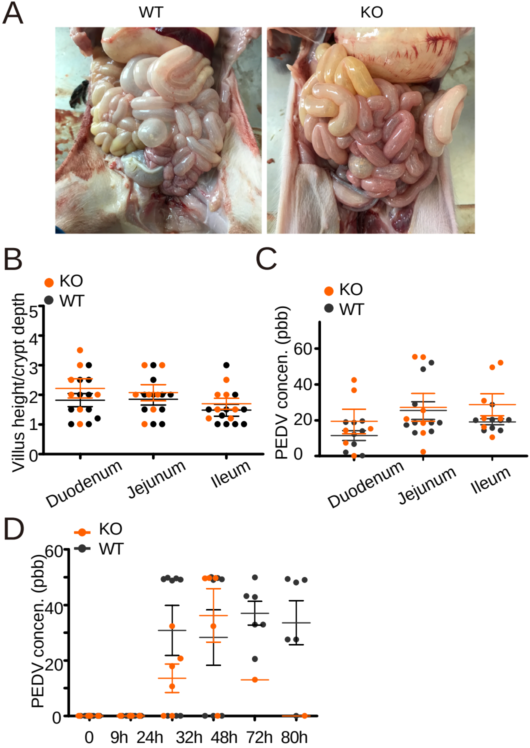
A
WT
KO
B
KO
WT
Villus height/crypt depth
0
1
2
3
4
5
Duodenum
Jejunum
Ileum
C
KO
WT
PEDV concen. (pbb)
0
20
40
60
Duodenum
Jejunum
Ileum
D
KO
WT
PEDV concen. (pbb)
0
20
40
60
0
9h
24h
32h
48h
72h
80h

Date of nuclear transfer	No. oocytes cultured	No. matured oocyte (%)	Type of embryos	No. embryos cultured	No. cleaved embryos (%)	Blastocyst rate (%)
Jan 11, 2018	1674	982 (58.7%)	PA	44	38 (86.4%)	8 (18.2%)
			SCNT	21	20 (95.2%)	9 (42.9%)
Jan 18, 2018	1691	1001 (59.2%)	PA	73	70 (95.9%)	52 (71.2%)
			SCNT	22	19 (86.4%)	9 (40.9%)

Parthenogenetic activation was performed as a control of oocyte quality. PA: parthenogenetic activated oocytes; SCNT: somatic cell nuclear transfer

Surrogate sow ID	No. embryos transferred	Pregnancy (d28)	Pregnancy (d50)	Piglets at birth	Birth weight	Note
CRMY01416801344	176	Yes	Yes	3	1.06kg, 0.46kg, 0.64kg	One piglet died in day 1
CRMY01416801381	176	Yes	No	0	—	Spontaneous abortion
CRMY01416801362	176	Yes	Yes	3	0.68kg, 0.5kg, 0.74kg	One piglet died in day 1
LLMY01416028366	176	No	No	0	—	—
CRMY01416801499	176	Yes	Yes	4	1.44kg, 1.14kg, 1.22kg, 1.6kg	—
YYMY01416670602	176	Yes	No	0	—	Spontaneous abortion
LLMY01416314510	202	Yes	Yes	4	1.4kg, 0.82kg, 1.0kg, 0.98kg	One dead fetus
CRMY01416801607	203	Yes	Yes	2	NA	—
LLMY01416030204	202	Yes	Yes	3	1.2kg, 0.72kg, 1.16kg	—
LLMY01416027910	203	Yes	Yes	2	0.92kg, 0.94kg	One piglet died in day 1
Summary	1866	9/10 (90%)	7/10 (70%)	21/1866 (1.13%)		

Animal ID	Sequence	Description
K1, K2, K3, K4, K5	Allele 1: GCCGAGCATG TCCCCCAGGC CCCCACGTCG<br>CCCACCATCA CCACCACA------//------AGGGCAAAAGCA<br>TCGTCCGCTT	△130bp
	Allele 2: GCCGAGCATG TCCCCCAGGC CCCCACGTCG<br>CCCACCATCA CCACCACAGC CGCCAc++++++//++++++CaaC<br>gTGacgCtGA G++++++//++++++CAcGtCGcc<br>cA+++++ACCaccAC aGCCgcCaCA CAACGCTGTT<br>GCCTGATTCC TACAACGTGA CGCTGAGACC CTACCTCACT<br>CCCAACGCGG ATGGCCTGTA CATCTTCAAG	+43bp，<br>mismatch18bp
K11, K20	Allele 1: GCCGAGCATG TCCCCCAGGC CCCCACGTCG<br>CCCACCATCA CCACCACAGC CGCCtgattC cT-------- aCAA--<br>CGTG acgCtGagAC --CCTACCtc actCGCTGTT GCCTGATTCC<br>TACAACGTGA CGCTGAGACC CTACCTCACT CgCtg------<br>TtGCCTGTA CATCTTCAAG	△18bp,<br>mismatch<br>22bp
	Allele 2: GCCGAGCATG TCCCCCAGGC CCCCACGTCG<br>CCCACCATCA CCACCACA------//------ACGCTGTT<br>GCCTGATTCC TACAACGTGA CGCTGAGACC CTACCTCACT<br>CCCAACGCGG ATGGCCTGTA CATCTTCAAG	△54bp
K12, K17, K19	Allele 1: GCCGAGCATG TCCCCCAGGC CCCCACGTCG<br>CCCACCATCA CCACCACAGC CGCCAcaACg<br>Tga++++GcCtAcA aCgtGaCGct G+++++AAC+tGacgC<br>tGCCTAC++++++//++++++CtA CAACGt++cGcT<br>GCCT++++++//++++++GcTgCC TACAACGTGA CGCTGAGACC<br>CTACCTCACT CCCAACGCGG ATGGCCTGTA CATCTTCAAG	+52bp，<br>mismatch25bp
	Allele 2: GCCGAGCATG TCCCCCAGGC CCCCACGTCG<br>CCCACCATCA CCACCACAGC CGCCATCACC TTGGACCAGA<br>GCAAGCCGTG GAACCGGTAC CGCCTACC-A CAACGCTGTT<br>GCCTGATTCC TACAACGTGA CGC------//------GG<br>ATGGCCTGTA CATCTTCAAG	△26bp
K14, K16, K18	Allele 1: GCCGAGCATG TCCCCCAGGC CCCCACGTCG<br>CCCACCATCA CCACCACAGC CGCCATCACC TTGGACCAGA<br>GCAAGCCGTG GAACCGGTAC CGCCTACCCA --ACGCTGTT<br>GCCTGATTCC TACAACGTGA CGCTGAGACC CTACCTCACT<br>CCCAACGCGG ATGGCCTGTA CATCTTCAAG	△2bp
	Allele 2: GCCGAGCATG TCCCCCAGGC CCCCACGTCG<br>CCCACCATCA CCACCACAGC CGCCATCACC TTGGACCAGA<br>GCAAGCCGTG GAACCGGTAC CGCCTACCCA CAACaacGTg<br>aCgc--------tgACGTGA CGCTGAGACC CTACCTCACT<br>CCCAACGCGG ATGGCCTGTA CATCTTCAAG	△8bp,<br>mismatch 9bp

Piglet	Fecal shedding (TGEV), ppb, by hours post inoculation							
ID	0	12h	24h	36h	48h	56h	72h	80h
KO4	0	0	0	0	1.33618	1.358652	9.729438	0.637303
KO5	0.190112	0.212584	0.212584	0.212584	0.619326	0.601348	7.983371	0.605843
KO16	0	ND	2.213115§					
KO17	0	ND	0§					
KO18	0	ND	2.382172	7.185792	0§			
KO20	0	ND	0	4.26932	0§			
WT6	0	0	4.113708	4.113708	14.00135	13.86652	0.477753	0.477753
WT7	0.073258	0	13.70022	13.89124§				
WT8	0.142921	0.354157	13.89124	13.56539§				
WT9	0.459775	0.354157	13.82157	13.5227	0.354157	5.75191§		
WT10	0	0.282247	0.477753	13.74517	0.477753	13.93843§		
WT16	0	ND	6.73668	7.317281	10.00342	9.986339	8.604714§	
WT17	0	ND	6.466872	2.184085	6.355874	11.20325§		
WT18	0	ND	0.495219	ND	7.28323§			
WT19	0	ND	1.331967	4.91291	0	8.911901§		
WT20	0	ND	9.465505§					

KO4-20: APN-null piglets; WT7-20: Wild-type piglets; ppb, parts per billion; ND, not determined; -, no result as pig euthanized; §, euthanized.
